# Construction of Hierarchical CuO/Cu_2_O@NiCo_2_S_4_ Nanowire Arrays on Copper Foam for High Performance Supercapacitor Electrodes

**DOI:** 10.3390/nano7090273

**Published:** 2017-09-15

**Authors:** Luoxiao Zhou, Ying He, Congpu Jia, Vladimir Pavlinek, Petr Saha, Qilin Cheng

**Affiliations:** 1 Key Laboratory for Ultrafine Materials of Ministry of Education, School of Materials Science and Engineering, East China University of Science and Technology, 200237 Shanghai, China; czlx1990@126.com (L.Z.); congpujia@163.com (C.J.); 2 Centre of Polymer Systems, Tomas Bata University in Zlin, nam. T. G. Masaryka 5555, 760 01 Zlin, Czech Republic; vpavlinek@seznam.cz (V.P.); saha@utb.cz (P.S.)

**Keywords:** copper oxide, nickel cobalt sulfide, hierarchical composite nanowires, supercapacitor, electrochemical properties

## Abstract

Hierarchical copper oxide @ ternary nickel cobalt sulfide (CuO/Cu_2_O@NiCo_2_S_4_) core-shell nanowire arrays on Cu foam have been successfully constructed by a facile two-step strategy. Vertically aligned CuO/Cu_2_O nanowire arrays are firstly grown on Cu foam by one-step thermal oxidation of Cu foam, followed by electrodeposition of NiCo_2_S_4_ nanosheets on the surface of CuO/Cu_2_O nanowires to form the CuO/Cu_2_O@NiCo_2_S_4_ core-shell nanostructures. Structural and morphological characterizations indicate that the average thickness of the NiCo_2_S_4_ nanosheets is ~20 nm and the diameter of CuO/Cu_2_O core is ~50 nm. Electrochemical properties of the hierarchical composites as integrated binder-free electrodes for supercapacitor were evaluated by various electrochemical methods. The hierarchical composite electrodes could achieve ultrahigh specific capacitance of 3.186 F cm^−2^ at 10 mA cm^−2^, good rate capability (82.06% capacitance retention at the current density from 2 to 50 mA cm^−2^) and excellent cycling stability, with capacitance retention of 96.73% after 2000 cycles at 10 mA cm^−2^. These results demonstrate the significance of optimized design and fabrication of electrode materials with more sufficient electrolyte-electrode interface, robust structural integrity and fast ion/electron transfer.

## 1. Introduction

Over the past few years, considerable effort has been made to search for clean, efficient and renewable energy sources due to the limited available fossil fuels and serious pollution problems caused by conventional energy technologies [1,2,3,4]. Many energy storage devices are thus being developed, with the aim of effective use of various energy sources. As one of the most promising energy storage systems, supercapacitors (SCs) have drawn intense attention because of their favorable characteristics including high power density, long cycle life, rapid charge-discharge process, and environmental friendliness [5,6,7]. Although considerable progress has been achieved so far in SC applications, they are still restricted by their lower energy density in comparison with secondary batteries. The kinetic characteristics and electrochemically active surface area of the electrode materials mainly influence the performance of SCs, thus the electrode materials with appropriate pore structure and superior electrical properties are perfectly reasonable [8]. So far, carbon-based materials, conducting polymers and transition metal oxides/hydroxides, are among the most extensively investigated electrode materials for SCs [9,10,11,12]. However, they have some obvious drawbacks, including low specific capacitance, poor cycling stability and low electronic conductivity, which inevitably deteriorate their electrochemical performance and restrict their applications in SCs. Hence, it is imperative to develop advanced composites combining synergistic contributions from single components to maximize the performance of electrodes.

In recent years, transition metal sulfides such as nickel sulfides [13,14], cobalt sulfides [15,16] and ternary nickel cobalt sulfides [17,18,19] have been explored as promising electrodes for SCs because of their higher electrical conductivity and richer redox reactions than those of their oxide and hydroxide counterparts [20]. Impressively, ternary metal sulfides also possess much higher electrical conductivity and exhibit better electrochemical performance as compared to binary metal sulfides. To date, various nanostructures of ternary metal sulfides have been developed and shown high capacitive performance in SCs applications. More attractively, 3D hierarchical nanoarchitectures on conductive substrates not only can effectively enhance the utilization of active materials, due to their large surface area and short electron- and ion-transport pathways, but also combine the advantages of both components [21,22,23,24]. As a result, the ternary metal sulfides based core-shell composite arrays demonstrate excellent performance. For instance, Gong et al. reported the in situ growth of NiCo_2_S_4_@Ni_3_V_2_O_8_ core/shell hybrid on Ni foam via a facile two-step process. The composite electrode exhibited a higher specific capacity of 512 C g^−1^ and a better rate capability of 396 C g^−1^ at 10 A g^−1^ [24]. Xie et al. also developed an effective strategy to fabricate hierarchical NiCo_2_S_4_@CoS*_x_* nanotube arrays on Ni foam, and it showed a high areal capacitance of 4.74 F cm^−2^ at a current density of 5 mA cm^−2^, a good rate capability (2.26 F cm^−2^ at 50 mA cm^−2^) and cycle stability (76.1% capacitance retention after 1500 cycles at 50 mA cm^−2^), which were superior to those of NiCo_2_S_4_ nanotubes [25]. Although a significant advance has been made in the fabrication of ternary metal sulfides based 3D core-shell composites, they still suffer from significant degradation during the fast and long-term cycling process due to structural or morphological failure, which greatly limits their electrochemical performance. Thus, the rational design and fabrication of hierarchical nanocomposites with a highly integrated style, mechanical robustness and efficient charge/mass transport have been the focus of research to optimize the overall performance of SCs.

On the other hand, copper oxides (CuO and Cu_2_O-based SCs have also recently attracted wide interest because of their abundance, low cost, non-toxicity and easy preparation of different nanostructures [26,27,28,29]. But the low electrical conductivity and poor cycling stability hinder their charge storage performance. To solve the disadvantages, one effective way is to incorporate pseudocapacitive materials into copper oxides to enhance their capacitance, particularly as a smart nanoarchitecture is constructed to facilitate the charge transfer process [30,31,32]. Based on the point, it is expected that CuO/Cu_2_O@NiCo_2_S_4_ composite nanowire arrays grown on copper foam may result in fascinating electrochemical performance. Yet for that, it is still challenging to bring cost-effectiveness to hierarchical CuO/Cu_2_O@NiCo_2_S_4_ based free-standing electrodes with outstanding performance.

Herein, we design and fabricate a free-standing, 3D hierarchical CuO/Cu_2_O@NiCo_2_S_4_ core-shell nanocomposite electrode based on nickel cobalt sulfides nanosheets grown on CuO/Cu_2_O nanowire arrays. CuO/Cu_2_O nanowire arrays are firstly grown on Cu foam by thermal oxidation, followed by one-step electrodeposition of NiCo_2_S_4_, which is directly used as binder-free electrode for electrochemical evaluation. The as-prepared composite electrode exhibits an ultrahigh specific capacitance of 3.186 F cm^−2^ at 10 mA cm^−2^, good rate capability and excellent cycling stability with a capacitance retention of 96.73% after 2000 cycles at 10 mA cm^−2^. In addition, the relationship between structural and electrochemical properties of the composites is also studied systematically. 

## 2. Experimental Section

### 2.1. Chemicals and Materials

All chemical agents were analytical grade and purchased from Sinopharm Chemical Reagents Co., Ltd. (Shanghai, China). Commercially Cu foam was purchased from Lizhiyuan Battery Materials Co., Ltd. (Shanxi, China).

### 2.2. Fabrication of CuO/Cu_2_O Nanowire Arrays on Cu Foam

Commercially, Cu foam (1.2 mm thick) was used as the substrates to grow 3D CuO/Cu_2_O nanowire arrays. Typically, the Cu foams were first immersed in ethanol solution followed by ultrasonic treatment for 20 min, and then dried at 80 °C for 12 h in a vacuum oven. Finally, they were thermally oxidized in a muffle furnace at 400 °C for 4 h with a heating rate of 3 °C min under air atmosphere.

### 2.3. Synthesis of CuO/Cu_2_O@NiCo_2_S_4_ Core-shell Nanostructures

The NiCo_2_S_4_ nanosheets with different Ni–Co–S composition were electrochemically co-deposition on the surface of CuO/Cu_2_O nanowires. Typically, the electrolyte solution contains 10 mM NiCl_2_·6H_2_O with different concentrations of CoCl_2_·6H_2_O (0, 5, 10, 15, 25 mM) and 0.75 M thiourea. The corresponding composite electrodes were designated as CuO/Cu_2_O@NiCo_2_S_4_-1, CuO/Cu_2_O@NiCo_2_S_4_-2, CuO/Cu_2_O@NiCo_2_S_4_-3, CuO/Cu_2_O@NiCo_2_S_4_-4, CuO/Cu_2_O@NiCo_2_S_4_-5, respectively. The electrodeposition was performed with a three-electrode system using CuO/Cu_2_O/Cu as the working electrode, Pt plate as the counter electrode, and Ag/AgCl as the reference electrode, respectively. The electrochemical reaction took place with cyclic voltammetry at a scan rate of 5 mV s^−1^ for 3 cycles within a voltage range of −1.2 to 0.2 V. The obtained composite electrodes were carefully rinsed with distilled water and ethanol and then dried at 60 °C under vacuum for 12 h. 

### 2.4. Material Characterization 

X-ray diffraction (XRD) patterns were recorded on a Rigaku D/MAX 2550 diffractometer (Rigaku Corporation, Tokyo, Japan) equipped with a Cu Kα radiation generator. The morphological characterization was analyzed by field emission scanning electron microscopy (FE-SEM, S-4800, Hitachi, Tokyo, Japan) equipped with an energy dispersive X-ray spectrometer (EDS) and high resolution transmission electron microscopy (HRTEM, JEM-2011, JEOL, Tokyo, Japan). X-ray photoelectron spectroscopy (XPS) was performed on a Thermo ESCALAB 250 X-ray photoelectron spectrometer (Thermo Scientific, Waltham, MA, USA) at a pressure of about 2 × 10^−9^ Pa using Al Kα X-ray as the excitation source. 

### 2.5. Electrochemical Measurements

The electrochemical properties of the as-obtained electrodes were investigated on a CHI 660E electrochemical workstation (Shanghai Chenhua Instrument Co., Shanghai, China) with a three-electrode cell in 3 M KOH aqueous electrolyte at room temperature. The composites electrodes, Pt plate and Ag/AgCl electrode were served as the working electrode, the counter electrode and the reference electrode, respectively. The applied potential window of cyclic voltammetry (CV) and galvanostatic charge–discharge (GCD) curves was set from −0.3 to 0.6 V and −0.1 to 0.4 V. The areal capacitance *C_s_* (F cm^−2^) is calculated by the equation: *Cs = IΔt/SΔV*, where *I* (A) is discharging current, Δ*t* (s) is the discharging time, *S* (cm^2^) is the area of the electrode and Δ*V* is the discharging potential window. The electrochemical impedance spectroscopy (EIS) was conducted within the frequency range between 0.01 to 100 kHz.

## 3. Results and Discussion

**Figure 1:**
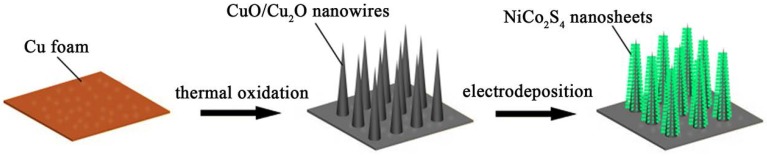
Schematic illustration of the CuO/Cu_2_O@NiCo_2_S_4_ core-shell nanowire arrays on Cu foam.

Figure 1 depicts the two–step preparation process of CuO/Cu_2_O@NiCo_2_S_4_ composite nanowire arrays on Cu foam. Firstly, CuO/Cu_2_O nanowires are directly grown on Cu substrate via one-step thermal oxidation of Cu foam, which has been confirmed by previous studies [33,34,35]. Two oxide layers on the Cu foam can be detected after thermal treatment: a very thin top layer of CuO and a bottom layer of Cu_2_O. Then, the CuO/Cu_2_O nanowire arrays are uniformly decorated with NiCo_2_S_4_ nanosheets by electrodeposition. The obtained composite possesses hierarchical core-shell nanoarchitectures which might show short ion transport/diffusion pathway and close contact between active materials and electrolyte.

**Figure 2:**
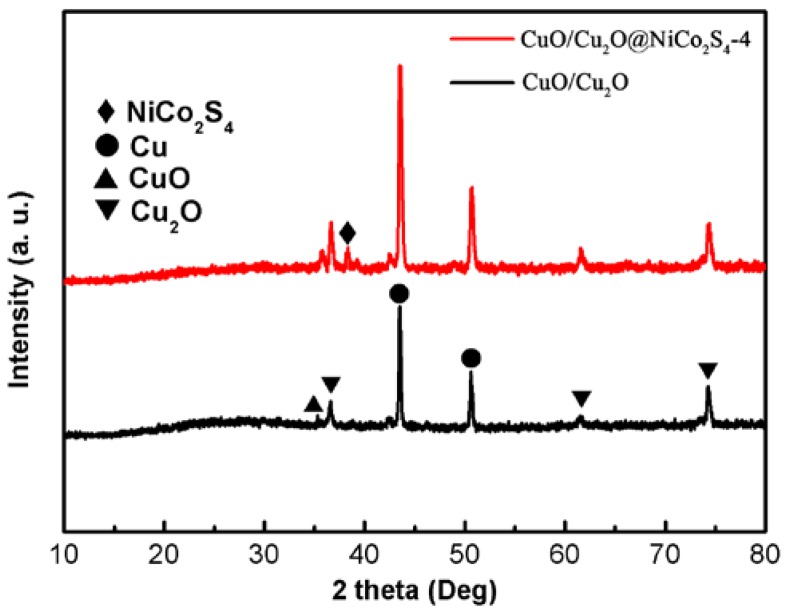
X-ray diffraction patters of the as–prepared CuO/Cu_2_O/Cu and CuO/Cu_2_O@NiCo_2_S_4_-4.

Typical XRD patters of the CuO/Cu_2_O and CuO/Cu_2_O@NiCo_2_S_4_-4 samples are given in Figure 2. Both distinctive peaks at 43.3° and 50.4° assigned to (111) and (200) planes of Cu (JCPDS No. 65–9026) [29] can be observed in the pattern of CuO/Cu_2_O/Cu composite, which indicates that Cu foam substrate is not entirely oxidized. The diffraction peaks at 36.5°, 61.5° and 74.6° are attributed to the crystal planes of cubic Cu_2_O (JCPDS No. 78–2076), and the peak at 35.6° is ascribed to (111) crystal plane of CuO (JCPDS No. 74–1021) [27,29]. Importantly, the above diffraction peaks all appear in the pattern of CuO/Cu_2_O@NiCo_2_S_4_-4 composite, but a new peak at 38.1° is consistent with (400) plane of cubic type NiCo_2_S_4_ (JCPDS No. 45–1477) [24,36], suggesting the poor crystallinity or small amount of NiCo_2_S_4_ obtained by electrodeposition. 

**Figure 3:**
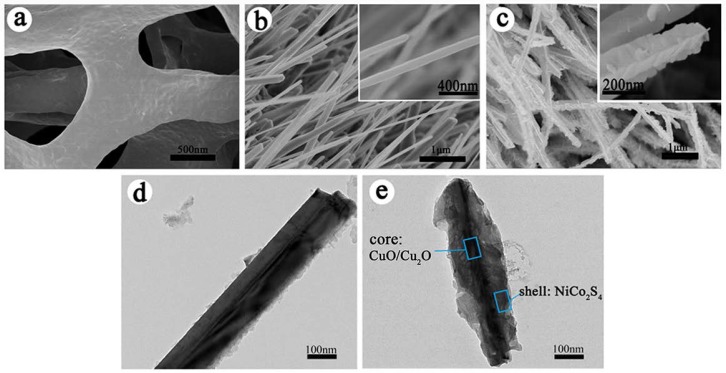
Scanning electron microscopy (SEM) images of (**a**) copper foam; (**b**) CuO/Cu_2_O nanowires and (**c**) CuO/Cu_2_O@NiCo_2_S_4_-4 composite nanowires (The insets in (**b**) and (**c**) show high-magnification of respective SEM images); transmission electron microscopy (TEM) images of (**d**) CuO/Cu_2_O nanowires and **(e**) CuO/Cu_2_O@NiCo_2_S_4_-4 composite nanowires.

In order to characterize the structure and surface morphology of hierarchical CuO/Cu_2_O@NiCo_2_S_4_-4 composite, scanning electron microscopy (SEM) and transmission electron microscopy (TEM) images are presented in Figure 3. As shown in Figure 3a, the copper foam shows a 3D open network framework, indicating its relatively high surface area and considerable electroactive sites for ion diffusion onto the nanowire surface. After thermal treatment of the Cu foam for some time, its skeleton is completely covered by dense nanowire arrays which are almost vertically grown on the Cu substrate (Figure 3b). The average length of CuO/Cu_2_O nanowires is around 5–10 μm and diameter is ~50 nm. After coating with NiCo_2_S_4_ nanosheets by the co-electrodeposition route, CuO/Cu_2_O@NiCo_2_S_4_ composites are obtained (Figure 3c). It is clear that NiCo_2_S_4_ nanosheets are grown uniformly on the CuO/Cu_2_O nanowires’ backbone, resulting in the hierarchical core-shell nanostructures on Cu foam. The core-shell nanostructure can be distinctly found and the average thickness of the NiCo_2_S_4_ shell is ~20 nm. It is worth noting that numerous voids between composite nanowires can be clearly observed, which facilitate the transport of ions between the electrode and electrolyte. The detailed structure of CuO/Cu_2_O@NiCo_2_S_4_ core-shell composite is also verified by TEM images. Figure 3d reveals the single CuO/Cu_2_O nanowire with smooth surface, while its surface becomes rough after coating with interconnected NiCo_2_S_4_ nanosheets (Figure 3e). And the width of the NiCo_2_S_4_ shell is ~100 nm. The rough and ultrathin nanosheets on the CuO/Cu_2_O nanowire core endow the composite with large surface area in favor of intercalation/de-intercalation of ions. In addition, CuO/Cu_2_O nanowires directly grown on Cu foam act as a wonderful scaffold for uniform growth of the metal sulfides nanoflakes, and hence the adhesion of the CuO/Cu_2_O@NiCo_2_S_4_ composite on Cu foam is very robust and the cycling stability of composite electrode is thus enhanced significantly. In order to further reveal the structural differences between CuO/Cu_2_O@NiCo_2_S_4_ composites obtained with various concentration ratio of CoCl_2_·6H_2_O to NiCl_2_·6H_2_O (i.e., ratio of Co^2+^/Ni^2+^), SEM images of these composites with various concentration ratio of Co^2+^/Ni^2+^ are now provided in Figure S1. It can be found that NiCo_2_S_4_ nanosheets on CuO/Cu_2_O nanowires become more evident and well defined hierarchical structures are thus formed with increasing concentration ratio of Co^2+^/Ni^2+^.

Energy-dispersive X-ray spectroscopy (EDS) mapping was carried out to clarify the elemental distribution of the NiCo_2_S_4_ nanosheet arrays. Figure S2b,c,e exhibit the elemental mapping of nickel (e), cobalt (b), sulfide (c), respectively. The even distribution of the Ni, Co, S elements reveals orderly growth of the NiCo_2_S_4_ nanosheets on the surface of CuO/Cu_2_O composite nanowires, which is more evidence that NiCo_2_S_4_ nanosheets have been synthesized successfully via electrodeposition.

**Figure 4:**
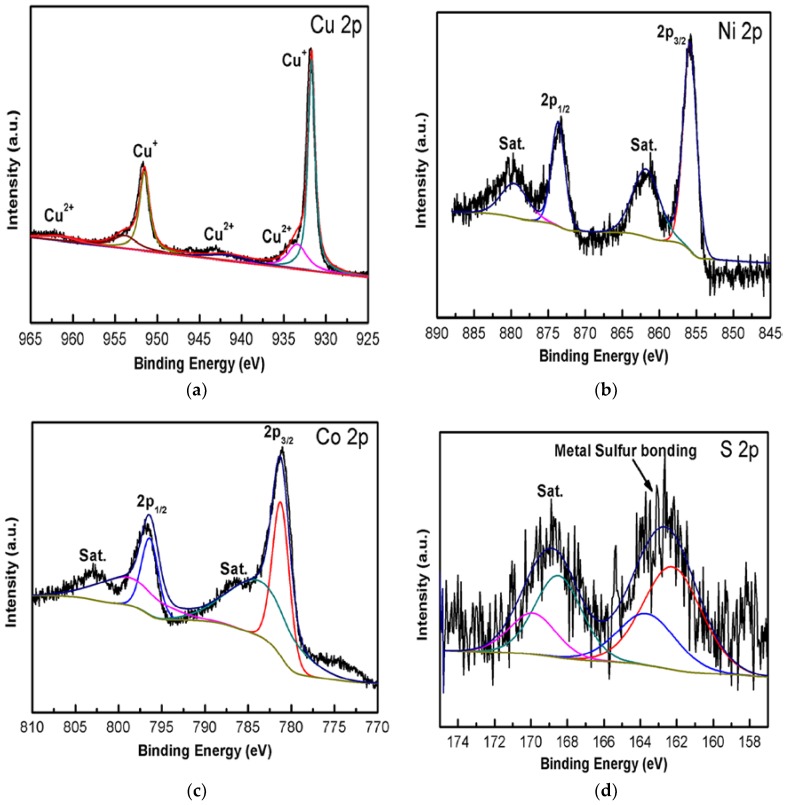
X-ray photoelectron spectroscopy (XPS) spectra of (**a**) Cu 2p; (**b**) Ni 2p; (**c**) Co 2p and (**d**) S 2p for the CuO/Cu_2_O@NiCo_2_S_4_-4 composite.

In order to analyse the elemental composition and chemical state in CuO/Cu_2_O@NiCo_2_S_4_ composite, XPS measurements were performed (Figure 4). As shown in Figure 4a, two typical peaks located at 931.7 eV and 952.0 eV correspond to Cu 2p_3/2_ and Cu 2p_1/2_, respectively, indicating existence of the Cu^+^ in the composite [37,38]. Moreover, the observed peaks at 943.0 eV and 962.5 eV imply the presence of unfilled Cu 3d^9^ shell of Cu^2+^ in the composite [39]. The Co 2p and Ni 2p spectra can be fitted with the two spin-orbit doublets and two shakeup satellite. Figure 4b presents the Ni 2p spectrum, the binding energies at around 875.2 eV and 857.0 eV are ascribed to Ni 2p_1/2_ and Ni 2p_3/2_, respectively, confirming the existence of Ni^2+^, while the peaks at 879.5 eV and 861.7 eV of Ni 2p are related to Ni^3+^ in the sample [40,41]. For the Co emission spectrum (Figure 4c), the observed peaks at 796.3 eV and 781.2 eV of Co 2p are characteristics of Co^2+^, whereas the peaks at 798.7 eV and 783.5 eV of Co 2p are well matched with Co^3+^ according to the previous reported [42]. As for the S 2p high resolution spectrum (Figure 4d), the binding energy at 162.1 eV can be attributed to the S 2p_3/2_ core level, which is a typical peak of the metal-sulfur bonds (Ni–S and Co–S bonding) in the Ni–Co sulfides [43] and the peak at 169 eV in S 2p is assigned to surface sulfur [44]. In the light of the XPS analysis, the surface of the composite consists of Ni^2+^, Ni^3+^, Co^2+^, Co^3+^ and S^2−^. The results are in agreement with previous studies on NiCo_2_S_4_ [45].

**Figure 5:**
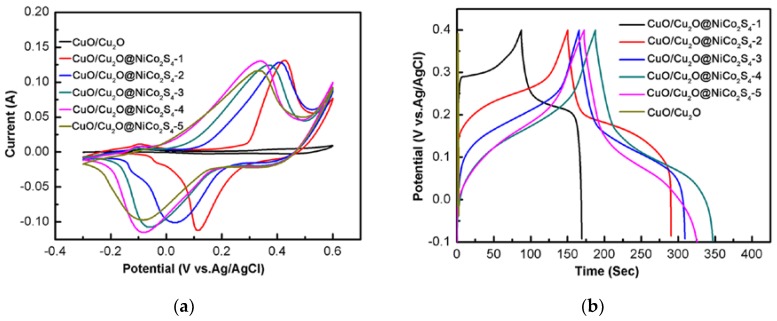

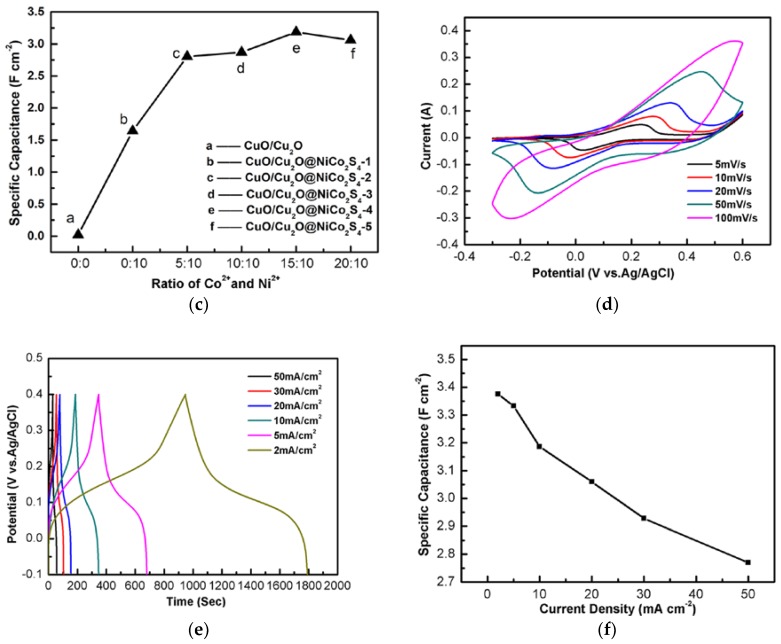
(**a**) Cyclic voltammetry (CV) curves at a scan rate of 20 mV s^−1^ and (**b**) galvanostatic charge–discharge (GCD) curves of all the CuO/Cu_2_O@NiCo_2_S_4_ electrodes at a current density of 10 mA cm^−2^; (**c**) Specific capacitance of CuO/Cu_2_O@NiCo_2_S_4_ composites as a function of ratio of Co^2+^/Ni^2+^ at a current density of 10 mA cm^−2^; (**d**) CV curves of the CuO/Cu_2_O@NiCo_2_S_4_-4 electrode; (**e**) GCD curves of the CuO/Cu_2_O@NiCo_2_S_4_-4 electrode; (**f**) specific capacitance of CuO/Cu_2_O@NiCo_2_S_4_-4 electrode at different current densities.

The electrochemical properties of the CuO/Cu_2_O@NiCo_2_S_4_ composites as binder-free electrodes were investigated in a standard three-electrode system. As can be seen from Figure 5a, all curves demonstrate a pair of well-defined redox peaks, showing the pseudocapacitive behavior of all composites. Clearly, the ternary composites possess the larger areas inside the CV curves than that CuO/Cu_2_O, demonstrating that incorporation of NiCo_2_S_4_ nanosheets result in larger capacitance of composites than CuO/Cu_2_O. Furthermore, the CuO/Cu_2_O@NiCo_2_S_4_-4 electrode has the largest CV integrated area among all composite electrodes at the same scan rate, suggesting the CuO/Cu_2_O@NiCo_2_S_4_-4 electrode exhibits the best capacitive properties. Note that along with the Co^2+^ concentration increasing, the anodic peaks shift to lower potential. This can be explained by the fact that the cobalt sulfides have a lower redox potential in comparison with nickel sulfides based on their inherent electrochemical response to the electrolyte [44]. Figure 5b depicts the GCD curves of composites with different ratio of Co^2+^ to Ni^2+^. Apparently, the longest discharging time of CuO/Cu_2_O@NiCo_2_S_4_-4 electrode exactly reflects its largest capacitance, as confirmed by the CV results. The specific capacitance of composites was calculated in terms of GCD curves and the results are plotted in Figure 5c. As the ratio of Co^2+^/Ni^2+^ is increased, the capacitance of the composites increases and reaches a maximum at Co^2+^/Ni^2+^ = 15/10 then decreases, that is, the CuO/Cu_2_O@NiCo_2_S_4_-4 electrode exhibits the largest capacitance of 3.186 F cm^−2^ at a current density of 10 mA cm^−2^. Therefore, CuO/Cu_2_O@NiCo_2_S_4_-4 is chosen as a representative of CuO/Cu_2_O@NiCo_2_S_4_ composites, and its structural and electrochemical properties are emphasized in our experiment.

Figure 5d shows the CV curves of CuO/Cu_2_O@ NiCo_2_S_4_-4 electrode at various scan rates of 5, 10, 20, 50, 100 mV s^−1^. The noticeable anodic and cathodic peaks in all CV curves indicate that the electrode materials obtained own Faradaic redox reactions and pseudocapacitive characteristics which is caused by the reversible reactions of Co^2+^/Co^3+^ and Ni^2+^/Ni^3+^ with OH^-^ according to the following equations [46]:

CoS + OH^−^ ↔ CoSOH + e^−^ (1)

CoSOH + OH^−^ ↔ CoS + H2O + e^−^ (2)

NiS + OH^−^ ↔ NiSOH + e^−^ (3)

Clearly, all the curves depict the similar redox peaks and the intensity of current increases with scan rate from 5 to 100 mV s^−1^. The anodic peaks shift to higher potentials and the cathodic peaks shift to lower potentials accordingly, indicating an enlarged potential window. Even as the scan rate increases up to 100 mV s^−1^, the redox peaks still retain, which indicates that the hierarchical structure is beneficial to faster Faradic reactions. Figure 5e presents the GCD curves of CuO/Cu_2_O@NiCo_2_S_4_-4 electrode at different current density. The similar symmetric potential-time curves demonstrate the high charge-discharge coulombic efficiency and low polarization of the 3D hierarchical composite electrode [47]. To further evaluate potential applications of the resulting composite electrodes for SCs, the rate capability of the CuO/Cu_2_O@NiCo_2_S_4_-4 electrode was also investigated by GCD measurements at various current densities (Figure 5f). The specific capacitance of this optimized electrode was calculated to be 3.376, 3.333, 3.186, 3.060, 2.928, 2.77 F cm^−2^ at 2, 5, 10, 20, 30, 50 mA cm^−2^, respectively. Although the capacitance decreases with increasing current densities, around 82.06% of initial capacitance is still retained as the current density increased from 2 to 50 mA cm^−2^. Good rate performance might be due to the synergistic effects of NiCo_2_S_4_ nanosheets and CuO/Cu_2_O nanowire arrays. CuO/Cu_2_O@NiCo_2_S_4_ core-shell nanowire arrays on Cu foam with high conductivity and specific surface area can not only facilitate the electrolyte access and exposure of the active sites to the electrolyte, but also effectively collect and transfer charges. In addition, the robust adhesion of composite nanowires on Cu foam also ensures its structural stability during the electrochemical reaction.

**Figure 6:**
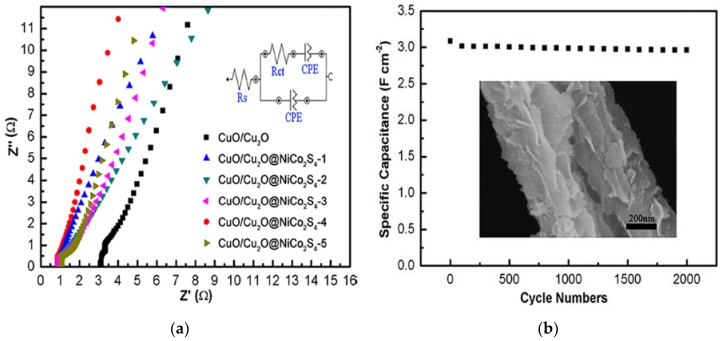
(**a**) Nyquist plots of all as-prepared electrodes, the inset is the equivalent circuit of CuO/Cu_2_O@NiCo_2_S_4_-4; (**b**) cycling performance of the CuO/Cu_2_O@NiCo_2_S_4_-4 electrode at a current density of 10mA cm^−2^ for 2000 cycles, the inset shows the SEM image of electrode after 2000 cycles.

To better understand the electrochemical performance of all the samples investigated, Nyquist plot was performed to investigate ion diffusion and charge transfer on the electrode/electrolyte interface, as shown in the Figure 6a. All the electrodes show a straight line in the low-frequency region. Ionic diffusion of electrolyte ions into active materials can be reflected by the slope of the straight line. It is evident that the largest slope of CuO/Cu_2_O@NiCo_2_S_4_-4 electrode reveals the best capacitive properties because of the fastest diffusion rate of electrolyte ions into the electrode materials. In the high-frequency region, the equivalent series resistance (*R_s_*) is indicated by intersection of the plots at the *X*-axis. Apparently, the CuO/Cu_2_O electrode exhibits the largest *R_s_* due to its highest resistance among all electrodes, while the CuO/Cu_2_O@NiCo_2_S_4_-4 electrode exhibits the lowest resistance (*R_s_* = 0.968 Ω), which indicates excellent capacitor behavior and low electrolyte diffusion resistance. Efficient attachment of NiCo_2_S_4_ nanoflakes on the CuO/Cu_2_O nanowire backbone offers an ideal pathway for electron transport. A small *R_s_* value facilitates rapid ion transport from solution to the NiCo_2_S_4_ nanosheets in electrolyte, leading to good rate capability of NiCo_2_S_4_-4, which can be also confirmed by Figure 5f.

The long-term stability of the electrode is also crucial for practical applications. Figure 6b shows the cycling performance of CuO/Cu_2_O@NiCo_2_S_4_-4 electrode at a current density of 10 mA cm^−2^. The specific capacitance decreases gently with the increasing cycle numbers. After 2000 cycles, the electrode could maintain a relatively high specific capacitance of 2.96 F cm^−2^, an outstanding capacitance retention of 96.73% is retained, indicating quite good cycling property, which is higher than other ternary sulfides, such as NiCo_2_S_4_ [48], NiCo_2_O_4_@Ni_3_S_2_ [49], and NiCo_2_S_4_@Ni(OH)_2_ [50]. To verify that this electrode material can give rise to considerable high capacitance retention, SEM imagery of the electrode material after 2000 cycles was conducted (Figure 6b, inset). The composite electrode can still maintain its 3D hierarchical structure even after 2000 cycles. The excellent electrochemical properties of CuO/Cu_2_O@NiCo_2_S_4_-4 can be caused by the following factors. Firstly, the composite electrode obtained exhibits hierarchical structure with the core (CuO/Cu_2_O nanowires) and the shell (NiCo_2_S_4_ nanosheets). The two parts can be both used as pseudocapacitive materials. The shell provides very large surface area to offer a number of electroactive sites for fast and reversible redox reactions to improve capacitance, while the core endows the active materials with robust adhesion on Cu foam to guarantee structural stability during cyclic process. Secondly, this hierarchical nanoarchitecture can provide superhighways for rapid electron transportation and ion diffusion between electrolyte and the electrode. Also, this hybrid structure can ensure a large contact surface between the electrode and electrolyte even at very high rate. Thirdly, the NiCo_2_S_4_ nanosheets directly grown on CuO/Cu_2_O nanowire arrays can create many open spaces and short ion diffusion paths in favor of the electrochemical reaction, and also avoid the addition of a polymer binder or additive in the electrode materials, ensuring good electrical conductivity. Therefore, the direct growth of NiCo_2_S_4_ nanosheets on CuO/Cu_2_O nanowires results in superior electrochemical performance of the ternary composite electrode.

## 4. Conclusions

In conclusion, the hierarchical NiCo_2_S_4_ nanosheets arrays grown directly on the CuO/Cu_2_O/Cu nanowires have been successfully constructed by electrodeposition for high performance SC electrode applications. Both the core (CuO/Cu_2_O nanowires) and the shell (NiCo_2_S_4_ nanosheets) act as the electroactive materials. Moreover, this hybrid structure also serves as a hierarchical scaffold for rapid ion diffusion and electron transport. The hierarchical CuO/Cu_2_O@NiCo_2_S_4_ electrode exhibits ultrahigh specific capacitance (2.77 F cm^−2^ at 50 mA cm^−2^), high rate performance (82.06% of capacitance retention from 2 to 50 mA cm^−2^) and excellent cycling stability (only 3.27% capacity loss after 2000 cycles at 10 mA cm^−2^). The experiment results not only stress the significance of optimal design and fabrication of electrode materials, but also propose a simple method for 3D electrode configuration in energy storage fields. 

## Supplementary Materials

The following are available online at http://www.mdpi.com/2079-4991/7/9/273/s1, Figure S1: SEM images of (a) CuO/Cu_2_O@NiCo_2_S_4_–1, (b)CuO/Cu_2_O@NiCo_2_S_4_–2, (c) CuO/Cu_2_O@NiCo_2_S_4_–3, (d) CuO/Cu_2_O@NiCo_2_S_4_–4, (e) CuO/Cu_2_O@NiCo_2_S_4_–5, Figure S2: EDS mapping of (a) O element, (b) Co element, (c) S element and (d) Cu elements (e) Ni element and (f) the cross section of CuO/Cu_2_O@ NiCo_2_S_4_–4.
