# Supplementary material for: Construction of Hierarchical CuO/Cu2O@NiCo2S4 Nanowire Arrays on Copper Foam for High Performance Supercapacitor Electrodes
Source: Nanomaterials (Basel). 2017 Sep 15;7(9):273. doi: 10.3390/nano7090273 (PMC5618384; doi:10.3390/nano7090273)
Supplement: Supplementary file 1 [file nanomaterials-07-00273-s001.pdf]

Supplementary Materials

Construction of hierarchical $CuO/Cu_2O@NiCo_2S_4$ nanowire arrays on copper foam for high performance supercapacitor electrodes

Luoxiao Zhou, Ying He, Congpu Jia, Vladimir Pavlinek, Petr Saha, Qilin Cheng

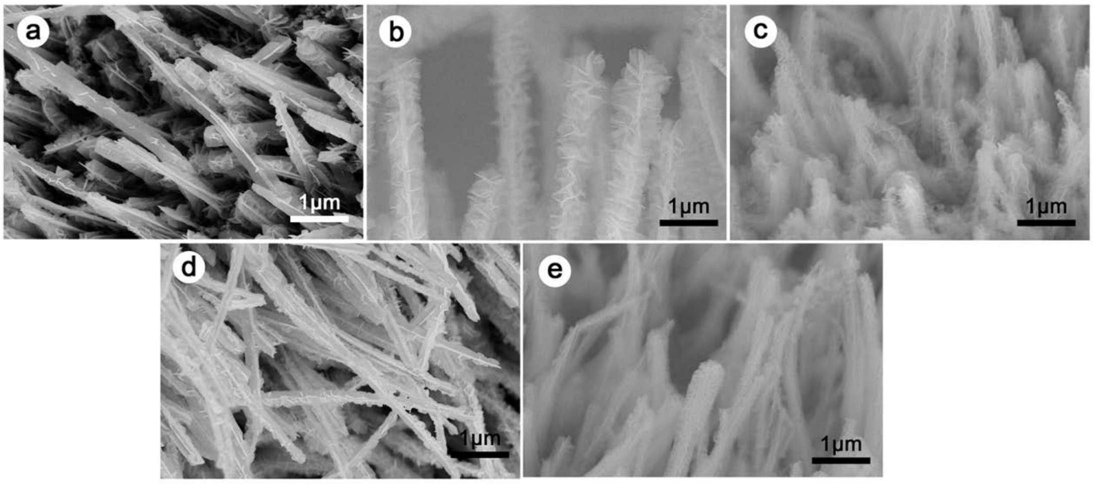

Figure S1. SEM images of (**a**) $CuO/Cu_2O@NiCo_2S_4$-1, (**b**)$CuO/Cu_2O@NiCo_2S_4$-2, (**c**) $CuO/Cu_2O@NiCo_2S_4$-3, (**d**) $CuO/Cu_2O@NiCo_2S_4$-4, (**e**) $CuO/Cu_2O@NiCo_2S_4$-5

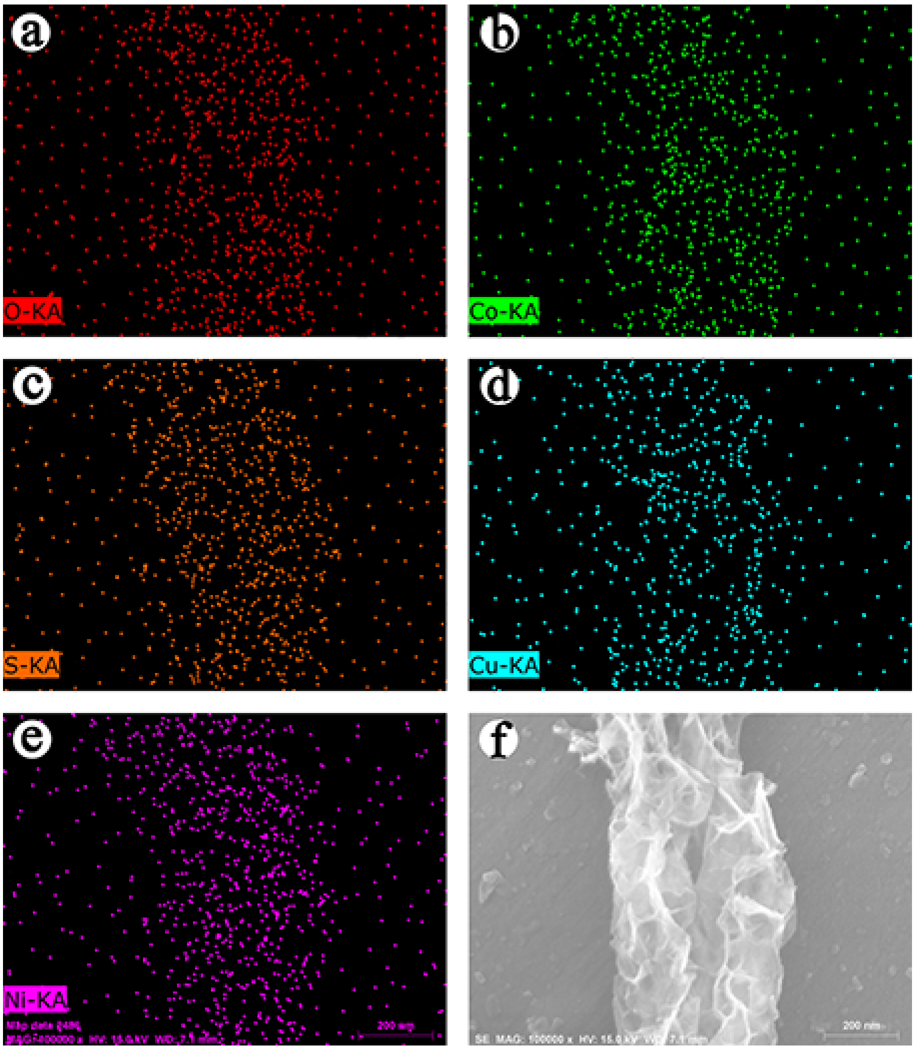

Figure S2. EDS mapping of (**a**) O element, (**b**) Co element, (**c**) S element and (**d**) Cu elements (**e**) Ni element and (**f**) the cross section of CuO/Cu_2O@ $NiCo_2S_4$-4.
